# The effect of influenza and pneumococcal vaccination in the elderly on health service utilisation and costs: a claims data-based cohort study

**DOI:** 10.1007/s10198-021-01343-8

**Published:** 2021-07-20

**Authors:** Josephine Storch, Carolin Fleischmann-Struzek, Norman Rose, Thomas Lehmann, Anna Mikolajetz, Srikanth Maddela, Mathias W. Pletz, Christina Forstner, Ole Wichmann, Julia Neufeind, Monique Vogel, Konrad Reinhart, Horst Christian Vollmar, Antje Freytag, the Vaccination 60+ Study Group

**Affiliations:** 1 Institute of General Practice and Family Medicine, Jena University Hospital, Bachstraße 18, 07743 Jena, Germany; 2 International Graduate Academy, Medical Faculty, Institute for Health and Nursing Science, Martin Luther University Halle-Wittenberg, Magdeburger Straße 8, 06112 Halle (Saale), Germany; 3 Center for Sepsis Control and Care, Jena University Hospital, Bachstraße 18, 07743 Jena, Germany; 4 Center for Clinical Studies, Jena University Hospital, Salvador-Allende-Platz 27, 07747 Jena, Germany; 5 Department for Anesthesiology and Intensive Care Medicine, Jena University Hospital, Am Klinikum 1, 07740 Jena, Germany; 6 Institute of Infectious Diseases and Infection Control, Jena University Hospital, Am Klinikum 1, 07747 Jena, Germany; 7 Department of Medicine I, Division of Infectious Diseases and Tropical Medicine, Medical University of Vienna, Währinger Gürtel, 18-20, 1090 Vienna, Austria; 8 Immunization Unit, Robert Koch Institute, Seestraße 10, 13353 Berlin, Germany; 9 Department of Anesthesiology and Intensive Care Medicine, BIH Visiting Professor/Charité Foundation, Charité Universitätsmedizin Berlin, Charitéplatz 1, 10117 Berlin, Germany; 10 Institute of General Practice and Family Medicine, Medical Faculty, Ruhr-University Bochum, Universitätsstraße 150, 44801 Bochum, Germany

**Keywords:** Claims data, Real-world data, Influenza vaccination, Pneumococcal vaccination, Elderly, Cost, Health care utilisation

## Abstract

**Background:**

To date, cost-effectiveness of influenza and pneumococcal vaccinations was assumed in several health economic modelling studies, but confirmation by real-world data is sparse. The aim of this study is to assess the effects on health care utilisation and costs in the elderly using real-world data on both, outpatient and inpatient care.

**Methods:**

Retrospective community-based cohort study with 138,877 individuals aged ≥ 60 years, insured in a large health insurance fund in Thuringia (Germany). We assessed health care utilisation and costs due to influenza- or pneumococcal-associated diseases, respiratory infections, and sepsis in 2015 and 2016. Individuals were classified into four groups according to their vaccination status from 2008 to 2016 (none, both, or either only influenza or pneumococcal vaccination). Inverse probability weighting based on 236 pre-treatment covariates was used to adjust for potential indication and healthy vaccinee bias.

**Results:**

Influenza vaccination appeared as cost-saving in 2016, with lower disease-related health care costs of − €178.87 [95% CI − €240.03;− €117.17] per individual (2015: − €50.02 [95% CI − €115.48;€15.44]). Cost-savings mainly resulted from hospital inpatient care, whereas higher costs occurred for outpatient care. Overall cost savings of pneumococcal vaccination were not statistically significant in both years, but disease-related outpatient care costs were lower in pneumococci-vaccinated individuals in 2015 [− €9.43; 95% CI − €17.56;− €1.30] and 2016 [− €12.93; 95% CI − €25.37;− €0.48]. Although we used complex adjustment, residual bias cannot be completely ruled out.

**Conclusion:**

Influenza and pneumococcal vaccination in the elderly can be cost-saving in selective seasons and health care divisions. As cost effects vary, interpretation of findings is partly challenging.

**Supplementary Information:**

The online version contains supplementary material available at 10.1007/s10198-021-01343-8.

## Introduction

Influenza and pneumococcal disease are important causes of death, morbidity, and money spent on related medical care worldwide [1]. Due to the gradual deterioration of their immune system (immunosenescence) and increasing comorbidity, the elderly are at increased risk for infections and infection complications. Therefore, national vaccination programs have a special focus on influenza and pneumococcal vaccination in the elderly [2, 3]. In Germany, seasonal influenza vaccination is recommended for individuals ≥ 60 years as well as for individuals with underlying medical conditions or close contact to patients at risk, independent of age. Also, for individuals ≥ 60 years, pneumococcal vaccination is recommended with a 23-valent pneumococcal polysaccharide vaccine (PPV). Booster vaccination every 6 years is recommended based on individual risk assessment [4]. For individuals with underlying chronic conditions associated with high risk for pneumococcal infection (e.g., immunosuppression, chronic liver disease, or renal failure), vaccination against pneumococci is recommended as a sequential immunization using a 13-valent pneumococcal conjugate vaccine (PCV) followed by PPV, and a booster vaccination every 6 years [4].

Germany has a multi-payer health care system with the statutory health insurance and the private health insurance based on different health insurance funds (HIF). The statutory health insurance covers about 88% of all German insurees [5]. It is financed by income-based insuree contributions to the single HIFs, in which individuals are insured. The single HIF in return covers costs for outpatient and inpatient health care services which also include influenza and pneumococcal vaccination. Single HIFs can act as major players in supporting nationwide and regional vaccination programs to increase vaccination rates. For informed decisions, they need reliable evidence on vaccine effectiveness as well as cost-effectiveness.

Cost-effectiveness of influenza and pneumococcal vaccination has been shown in numerous health economic simulation models, mostly reporting on costs per quality-adjusted life years gained based on parameter estimates from the literature or national data sources. A systematic review from Shields et al. showed that seven of the eight included studies identified influenza vaccination as cost-effective in the elderly in at least one scenario [6]. Systematic reviews from Porchia et al. and Dirmesropian et al. reported generally favourable results on the cost-effectiveness of pneumococcal vaccination [7, 8]. According to Porchia et al., all included studies confirm that vaccinating with PPV or PCV is cost-effective in individuals ≥ 60 years and in the general population [7]. Dirmesropian and colleagues found that nine out of ten studies considered PCV in the elderly as cost-effective, from which two studies valued PCV even as cost-saving [8]. However, all reviews pointed out that results of cost-effectiveness studies have to be interpreted cautiously [6–8]. The authors criticise the lacking or deficient reporting of data and underlying assumptions (e.g., herd immunity and overestimated duration of vaccine protection), the insufficient consideration of disease complications and associated diseases, as well as more general aspects of methods and design [6–8]. Due to these aspects and the fact that central effect parameters in modelling studies often do not originate from empirical data but rather from expert panels, the robustness of the results is questionable [6, 8]. In consequence, adequate parameter selection for health economic modelling studies evaluating vaccine effects is complex and a limiting factor [9].

On the contrary, evidence from real-world data on cost effects is much scarcer and except for a single pure health care utilisation (no cost) study [10], there are no European, and above all no German studies: A US study based on administrative claims data from 1990–1996 found that influenza vaccination of healthy and at-risk seniors was associated with substantial health benefits, cost-effectiveness, and even cost savings [11]. A Canadian population-based study using claims data from 1992 to 2014 showed reduced hospitalisation costs due to pneumonia after implementing a publicly funded pneumococcal vaccination program among all age groups, especially in the elderly [12]. A Japanese study observing a representative sample ≥ 75 years from 2008 found lower inpatient expenditure due to respiratory diseases, chronic heart failure, and other pre-defined diagnoses in influenza- and pneumococci-vaccinated individuals compared to those not vaccinated, but higher inpatient expenditure due to chronic obstructive pulmonary disease among vaccinees [13]. Claims data-based studies about vaccination effects on health care utilisation without measuring cost effects are less scarce. Their results will be compared with the study results at hand in the discussion section.

Health economic studies based on claims can generate evidence for vaccination cost effects from real-world data if they effectively limit the risks of biased effect estimation due to confounding variables as common issues of observational vaccination studies in the elderly [14, 15]. In such they can enlarge the body of evidence to support informed decision making. As the study was performed from a health insurers’ perspective, the results might be useful for decisions of HIF on their future engagement in vaccination programs, which aim to increase vaccination rates in elderly. To the best of our knowledge, internationally, there are no health economic studies using both inpatient and outpatient claims data for analysing cost effects of influenza and pneumococcal vaccination in the elderly, and for Germany, there is no claims data-based cost study at all. Therefore, the aim of the study was to estimate inpatient and outpatient health care utilisation and costs of influenza und pneumococcal diseases with and without vaccination against influenza and/or pneumococci.

## Methods

### Study design

We performed a cost analysis within a retrospective cohort study design with adjusted group-wise comparisons between unvaccinated and vaccinated individuals to investigate influenza, pneumococcal, and combined vaccination effects as relative difference (RDiff) on health care utilisation and costs of health care in elderly individuals ≥ 60 years in 2015 and 2016. This study is part of the vaccination60 + project, which was designed to increase influenza vaccination rates in individuals ≥ 60 years in Thuringia as a model region in Germany [16].

### Data source and study population

Claims data of a large German statutory HIF (AOK PLUS) covering a 9 year period were used to conduct the analysis. The AOK PLUS is the largest HIF in Central Germany and currently covers about 50% of its population. Claims data were derived from the billing data for all divisions of outpatient and inpatient health care services. They include a unique identification number for each insurant enabling longitudinal analysis. We included individuals who were ≥ 60 years on January 01, 2014 and were continuously insured with the HIF between 2008 and 2016 or died after 2014 (*n* = 209,703). Data from 2008 to 2014 were used to identify prior vaccination status and risk factors. Health care utilisation and cost effects were analysed in 2015 and 2016.

Ethical approval was obtained from the local legal authorities for research on human beings. As pseudonymous claims data need permission in accordance with §75 Social Security Code X, we applied for and received approval from the responsible regulatory authority. The design, performance, and report of the claims data analysis were based on the recommendations STROSA (A Consensus German Reporting Standard for Secondary Data Analyses) [17] and RECORD (Reporting of studies Conducted using Observational Routinely-collected health Data) [18].

**Fig. 1:**
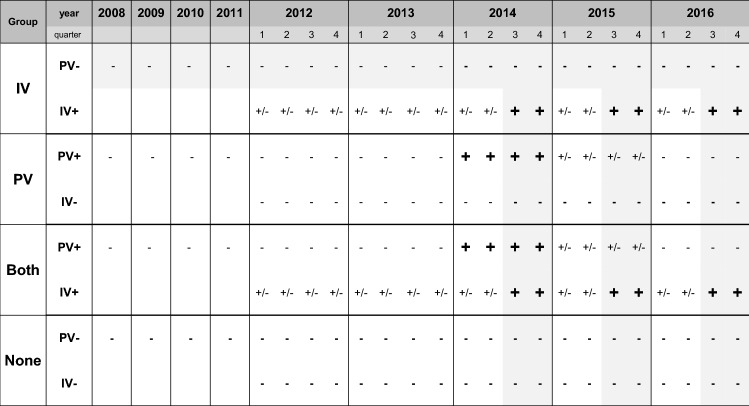
Vaccination scheme for insurant inclusion. IV: individuals who were vaccinated against influenza in the third or fourth quarter of the years 2014–2016 and not vaccinated against pneumococci in 2008–2016, PV: individuals with an initial pneumococcal vaccination in 2014 and no influenza vaccination in 2012–2014. BOTH: individuals with an initial pneumococcal vaccination in 2014 and with influenza vaccination in the third or fourth quarter of the years 2014–2016. NONE: Individuals who were not vaccinated against influenza in 2012–2016 nor against pneumococci in 2008–2016 were included in the control group. To avoid bias due to long-term vaccination effects, we excluded individuals with an additional pneumococcal vaccination in 2016 from PV and BOTH. To rule out biased estimations of vaccine effectiveness due to prior vaccinations, we also excluded individuals with prior pneumococcal vaccination in 2008–2013 in all groups and with prior influenza vaccination in 2012–2013 in PV and NONE. Outcomes were observed for 2015 and 2016.

To analyse single as well as additive effects of the vaccinations, individuals were included in four groups following a pre-defined vaccination scheme (see Fig. 1): individuals who were vaccinated against influenza in the third or fourth quarter of the years 2014–2016 and not vaccinated against pneumococci in 2008–2016 (IV), individuals with an initial pneumococcal vaccination in 2014 and no influenza vaccination in 2012–2016 (PV) and individuals with an initial pneumococcal vaccination in 2014 and with influenza vaccination in the third or fourth quarters of the years 2014–2016 (BOTH). Individuals who were not vaccinated against influenza in 2012–2016 nor against pneumococci in 2008–2016 were included in the control group (NONE). To avoid bias due to long-term vaccination effects, we excluded individuals with an additional pneumococcal vaccination in 2016 from PV and BOTH. To rule out biased estimations of vaccine effectiveness due to prior vaccinations, we also excluded individuals with prior pneumococcal vaccination in 2008–2013 in all groups and with prior influenza vaccination in 2012–2013 in PV and NONE.

### Outcome measure

Total direct costs associated with influenza or pneumococcal-associated diseases, respiratory infections, and sepsis in 2015 and 2016 (disease-related costs) were measured from a health insurers’ perspective for (i) hospital inpatient care, (ii) outpatient care, (iii) emergency services resulting in hospital admissions, (iv) inpatient rehabilitation, (v) drug prescriptions, and (vi) treatments (e.g., physical therapy and occupational therapy). Influenza- or pneumococcal-associated diseases, respiratory infections, and sepsis were defined via ICD-10-GM codes for influenza-like-illness (J09, J10, J11), acute respiratory infections (J00, J01, J02, J03, J04, J05, J06, J20, J21, J22, J44.0, B34.9), pneumonia (J12, J13, J14, J15, J16, J17, J18, U69.00), invasive pneumococcal disease (A40.3, J86, B95.3, G00.1), and sepsis (A02.1, A20.0, A20.7, A21.7, A22.7, A24.1, A26.7, A28.2, A32.7, A39.1, A39.2, A39.3, A39.4, A40, A41, A42.7, A48.3, A49.9, A54.8, B00.7, B37.6, B37.7, B49, R65.0, R55.1, R57.2).

We calculated health care utilisation per insurant with corresponding medical costs in the above-mentioned health care divisions if there was at least one defined: (i) primary or secondary inpatient diagnosis, (ii) confirmed outpatient diagnosis, (iii) primary or secondary inpatient diagnosis with a previous emergency case, and (iv) inpatient rehabilitation diagnosis. To measure (v) drug prescriptions associated with defined diseases, we calculated health care utilisation and costs for antivirals (ATC J05AC, J05AH) as well as antibiotics (ATC J01A, J01C, J01D, J01F, J01G, J01M, J01R, J01X, J02) in combination with at least one outpatient diagnosis for influenza-like-illness or pneumonia. Treatments (e.g., physical therapy) (vi) were defined as disease-related if they were prescribed with an indication for respiratory disorders.

Furthermore, we calculated total health care utilisation and costs of health care per insurant regardless of pre-defined diagnoses. Here, we assessed total costs as the sum of costs for outpatient care, hospital care (including inpatient and outpatient hospital care), emergency services, inpatient rehabilitation, outpatient drug prescriptions, therapeutic aids prescriptions, treatments (physiotherapy, occupational therapy, logopaedic, and podologist therapy) and nursing care, with separately reporting costs for outpatient care and hospital inpatient care, based on billed cases.

To consider intervention costs, we calculated total disease-related costs and total costs under addition of costs for vaccine drugs: As in Germany, costs for influenza and pneumococcal vaccine preparations are reimbursed by lump-sums to vaccinating physicians instead of per-patient, costs for vaccine doses are not included in the claims data. In contrast, remuneration costs for vaccination services are included in costs for outpatient care. That is why, costs for vaccination services did not have to be added. We estimated the mean amount of one vaccine dose by building the average of all influenza and pneumococcal vaccine doses, respectively, reimbursed by AOK PLUS in Thuringia. In 2015, mean costs of one influenza and pneumococcal vaccine dose were €8.47 and €60.24 and in 2016, €14.96 and €58.77, respectively. According to the number of billed vaccination services we included costs for influenza vaccine in IV, for pneumococcal vaccine in PV and for influenza and pneumococcal vaccine in BOTH. Assuming a duration of pneumococcal vaccination protection of 6 years [19], we added a sixth of the related costs (vaccine costs of €60.10 and remuneration cost of €6.00 per vaccination service) to total disease-related and total costs as well as to costs for outpatient care in 2015 as well as 2016.

Individuals who died during the follow-up period (2015, 2016) were included in the analyses with their health care utilisation and costs until death. For each follow-up year, we measured outcomes in individuals who were still alive at the beginning of the year.

### Statistical analysis

To control for systematic differences between the four groups, we used inverse probability weighting based on generalized propensity scores (GPS). Individual weights based on the GPS were estimated using a non-parametric tree-based generalized boosted regression model (GBRM), which allows for a large number of pre-treatment variables and accounts for possible higher order interactions among covariates in predicting group membership [20, 21]. We conducted an explorative literature search and gathered expert opinion to create a set of finally 236 pre-treatment covariates. These covariates included individual risk factors like comorbidities, long-term care, prior influenza-like-illness, pneumonia, and sepsis, as well as previous health care utilisation. A detailed description of the inverse probability weighting method we applied is published by Rose et al. [22] and covariates definition is shown in the supplementary material, Table S1.

Adjusted mean differences in health care utilisation and costs between vaccinated and unvaccinated individuals were tested for each follow-up year using the general linear model for complex data (PROC SURVEYREG in SAS 9.4) with inverse probability of treatment weighting [23]. Dummy variables for the three groups of vaccinated individuals (IV, PV, and BOTH) were used as predictor variables in the model. Unvaccinated individuals (NONE) served as the reference group. Hence, the regression coefficients represent the adjusted mean differences between each vaccination group and the control group. 95% Confidence interval (CI) was calculated using robust standard error obtained by the Taylor linearization method. The relative difference (RDiff), as the average percentage reduction in the outcome of each single vaccination group compared to NONE, was calculated as follows: RDiff $\frac{{\beta \times 100}}{{\bar{x}}}$, with $\bar{x}$ the adjusted mean of the reference group (NONE). Negative values of RDiff indicate a reduction in health care utilisation or costs.

## Results

**Fig. 2:**
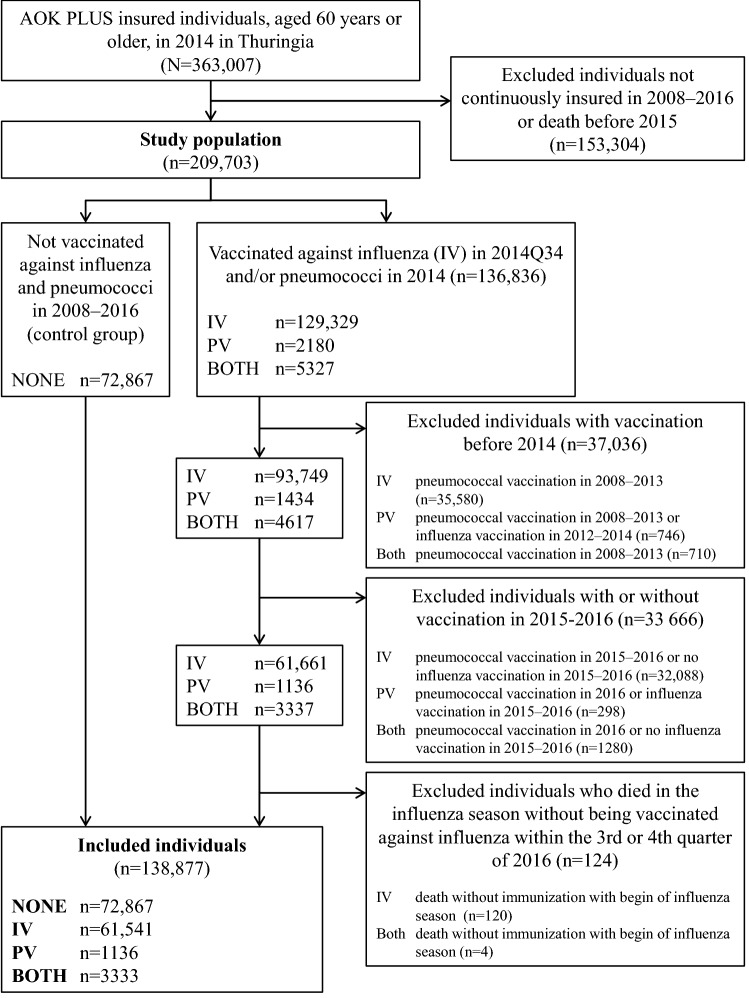
Flowchart of the study population inclusion

In total, 138,877 individuals were included as study population (Fig. 2). 61,541 individuals were only vaccinated against influenza (IV), 1136 only against pneumococci (PV), 3333 had received vaccines against both diseases (BOTH) and 72,867 individuals were unvaccinated (NONE). At the beginning of 2016, individuals still alive were *n* = 55,803 (IV), *n* = 1088 (PV), *n* = 3094 (BOTH), and *n* = 68,848 (NONE).

Individuals in the PV group were on average youngest, had a low number of comorbidities, were less often living in nursing homes, and had the lowest incidence of influenza-like-illness, pneumonia, and sepsis cases in 2013, among all vaccination groups (Table 1). Individuals in IV were on average oldest, had the highest number of comorbidities and underlying chronic conditions, were most often living in nursing homes, and had the highest number of outpatient general practitioner visits. Unvaccinated individuals were at a similar age as individuals vaccinated against both infections, but had the lowest comorbidities and underlying conditions as well as the lowest prior health care utilisation, and pneumonia and sepsis rates among all groups. Individuals in BOTH were younger, nevertheless with similar comorbidities like individuals in IV, but higher rates of incident diseases in 2013 before vaccination. After inverse probability weighting based on generalized propensity scores, the pairwise absolute standardized mean differences of baseline characteristics and covariates between all four groups were substantially reduced and below the critical value of 0.2 (Figure S1, supplementary material), indicating a good balance [24].

**Table 1:** Sample characteristics at baseline before weighting

	IV (*n* = 61,541)	PV (*n* = 1136)	BOTH (*n* = 3333)	NONE (*n* = 72,867)	Overall (*n* = 138,877)
Age, mean (SD)	76.94 (7.94)	69.62 (7.83)	72.87 (8.91)	72.38 (8.61)	74.39 (8.63)
Male sex, %	39.12	43.84	43.53	42.22	40.89
Charlson Comorbidity Index in 2013 (%)					
CCI: 0	17.38	34.15	19.92	39.63	29.25
CCI: 1	22.54	25.35	23.43	22.91	22.78
CCI: 2–4	46.12	33.54	43.68	30.91	37.98
CCI: 4	13.96	6.95	12.96	6.55	9.99
Underlying chronic conditions and comorbidity in 2013 (%)					
Chronic immunosuppression	19.88	13.47	17.91	13.20	16.27
Heart disease	64.32	43.31	58.36	42.18	52.39
Lung disease	25.60	19.10	27.87	17.76	21.49
Renal disease	24.37	16.73	25.26	14.50	19.15
Metabolic disease	78.80	67.25	76.06	58.05	67.75
Neurological disorders	33.07	21.92	29.40	19.87	25.96
Health care use in 2013					
Nursing home residence, %	8.62	1.76	7.38	1.94	5.03
Number of hospitalisations, mean (SD)	0.57 (1.10)	0.41 (0.95)	0.57 (1.13)	0.41 (1.01)	0.48 (1.05)
Number of GP outpatient visits, mean (SD)	13.63 (7.36)	9.77 (7.05)	13.08 (7.52)	8.53 (6.94)	10.91 (7.57)
Number of specialist outpatient visits, mean (SD)	11.81 (14.79)	9.38 (11.68)	12.49 (16.98)	7.63 (11.86)	9.62 (13.54)
Incident diseases in 2013, %					
Influenza (≥ 1 hospital- or outpatient diagnosis)	0.39	0.26	0.66	0.39	0.40
Pneumonia (≥ 1 hospital- or outpatient diagnosis)	3.29	2.46	4.29	1.93	2.59
Sepsis (≥ 1 hospital diagnosis)	1.80	1.23	1.86	1.16	1.46

### Impact of influenza vaccination (NONE vs. IV)

Comparing individuals only vaccinated against influenza with unvaccinated individuals (Table 2 and Table 3), we found lower disease-related total direct costs of − €178.87 [95% CI − €240.03; − €117.17] per patient in 2016, indicating an RDiff of -27.45%. Furthermore, we observed significantly lower costs for disease-related hospital inpatient care of − €71.09 ([95% CI − €135.21; − €6.98], RDiff − 12.73%) in 2015, and of − €193.38 ([95% CI − €252.71; − €134.06], RDiff − 32.30%) in 2016, as well as lower costs for disease-related emergency services in both years of follow-up (2015: − €1.53 [95% CI − €2.94; − €0.12], RDiff − 10.58%; 2016: − €2.05 [95% CI − €3.27; − €0.83], RDiff -16.31%). Despite this, there were statistically significant higher costs for disease-related outpatient care of €14.40 ([95% CI €5.45; €23.34], RDiff 40.85%) in 2015. The latter comes along with very small and thus practically not relevant but significantly higher costs for treatments (e.g., physical therapy) with indication for respiratory disorders in 2015 (€0.05 [95% CI €0.02; €0.08], RDiff 131.21%).

**Table 2:** Costs of health care: effect of influenza vaccination, per insurant in 2015 (NONE vs. IV)^a^

Direct costs	Adjusted mean costs in € (SE)		Adjusted mean Cost difference in € (95% CI)	Relative difference (RDiff)	p value
	NONE (*n* = 72,867)	IV (*n* = 61,541)			
Disease-related direct costs					
Total	608.95 (21.98)	558.93 (25.15)	− 50.02 (− 115.48; 15.44)	− 8.21%	0.13
Costs of disease-related hospital inpatient care	558.59 (21.61)	487.50 (24.56)	**− 71.09** (− 135.21; − 6.98)	**− 12.73%**	**0.03**
Costs of disease-related outpatient care	35.24 (2.70)	49.64 (3.68)	**14.40** (5.45; 23.34)	**40.85%**	** ≤ 0.01**
Costs of disease-related emergency services	14.44 (0.47)	12.91 (0.54)	**− 1.53** (− 2.94; − 0.12)	**− 10.58%**	**0.03**
Costs of disease-related antibiotics	0.52 (0.05)	0.59 (0.20)	0.07 (− 0.33; 0.47)	13.68%	0.73
Costs of disease-related inpatient rehabilitation	0.11 (0.06)	0.30 (0.11)	0.19 (− 0.05; 0.42)	168.08%	0.11
Costs of treatments with indication for respiratory disorders	0.04 (0.01)	0.08 (0.01)	**0.05** (0.02; 0.08)	**131.21%**	** ≤ 0.01**
Costs of antivirals	0.02 (0.004)	0.01 (0.002)	− 0.01 (− 0.02; 0.0005)	− 49.81%	0.06
Total direct costs					
Total	7325.04 (58.10)	7289.27 (73.37)	− 35.77 (− 219.20; 147.67)	− 0.49%	0.70
Costs of hospital inpatient care	2535.10 (33.93)	2262.31 (37.06)	**− 272.80** (− 371.29; − 174.31)	**− 10.76%**	** ≤ 0.01**
Costs of outpatient care	773.39 (10.18)	905.33 (11.64)	**131.94** (101.64; 162.25)	**17.06%**	** ≤ 0.01**

^a^Value rounded up to two decimal places or first identifiable digit

**Table 3:** Costs of health care: effect of influenza vaccination, per insurant in 2016 (NONE vs. IV)^a^

Direct costs	Adjusted mean costs in € (SE)^a^		Adjusted mean cost difference in € (95% CI)	Relative difference (RDiff)	p value
	NONE (*n* = 68,848)	IV (*n* = 55,803)			
Disease-related direct costs					
Total	651.60 (24.55)	472.73 (19.26)	**− 178.87** (-240.03; -117.71)	**− 27.45%**	** ≤ 0.01**
Costs of disease-related hospital inpatient care	598.72 (23.87)	405.34 (18.62)	**− 193.38** (-252.71; -134.06)	**− 32.30%**	** ≤ 0.01**
Costs of disease-related outpatient care	39.12 (3.69)	41.83 (3.25)	2.71 (-6.93; 12.36)	6.93%	0.58
Costs of disease-related emergency services	12.55 (0.39)	10.50 (0.49)	**− 2.05** (-3.27; -0.83)	**− 16.31%**	** ≤ 0.01**
Costs of disease-related antibiotics	0.44 (0.07)	0.34 (0.02)	− 0.10 (-0.25; 0.04)	− 23.61%	0.16
Costs of disease-related inpatient rehabilitation	0.72 (0.25)	0.41 (0.17)	− 0.31 (-0.89; 0.28)	− 42.67%	0.31
Costs of treatments with indication for respiratory disorders	0.05 (0.01)	0.07 (0.01)	0.02 (-0.01; 0.05)	33.05%	0.26
Costs of antivirals	0.003 (0.001)	0.005 (0.002)	0.002 (-0.002; 0.01)	55.58%	0.40
Total direct costs					
Total	7658.85 (62.37)	7201.73 (63.05)	**− 457.13** (− 630.94; − 283.31)	**− 5.97%**	** ≤ 0.01**
Costs of hospital inpatient care	2654.44 (36.92)	2114.92 (32.80)	**− 539.52** (− 636.32; − 442.73)	**− 20.33%**	** ≤ 0.01**
Costs of outpatient care	814.15 (11.27)	956.05 (13.50)	**141.90** (107.44; 176.36)	**17.43%**	** ≤ 0.01**

^a^Value rounded up to two decimal places or first identifiable digit

As displayed in Table 2 and Table 3, total costs were − €457.13 [95% CI − €630.94; − €283.31] lower per individual in 2016 (RDiff -5.97%). We further found significantly lower total costs for hospital inpatient care in both years of follow-up (2015: − €272.80 [95% CI − €371.29, − €174.31], RDiff -10.76%; 2016: − €539.52 [95% CI − €636.32; − €442.73], RDiff -20.33%), as well as higher total costs for outpatient care of €131.94 ([95% CI €101.64; €162.25], RDiff 17.06%) in 2015 and of €141.90 ([95% CI €107.44; €176.36], RDiff 17.43%) in 2016.

With regard to health care utilisation, there were systematic differences between vaccinated and unvaccinated individuals which are reflected in significant cost differences between groups described above (Table S2 and S3, supplementary material). We found a lower number of inpatient hospital cases in 2015 and 2016 (2015: -0.005 [95% CI -0.01; -0.001], RDiff -7.45%; 2016: -0.01 [95% CI -0.02; -0.01], RDiff -18.58%) and emergency services (2015: -0.01 [95% CI -0.01; -0.002], RDiff -10.39%; 2016: -0.01 [95% CI -0.01; -0.003], RDiff -15.99%). There was a higher health care utilisation regarding disease-related outpatient care in 2015 and 2016 (2015: 0.03 outpatient contacts [95% CI 0.02; 0.04], RDiff 11.21%; 2016: 0.02 [95% CI 0.01; 0.03], RDiff 9.62%) and treatments in 2015 (0.0005 [95% CI 0.0002; 0.001]; RDiff 94.80%).

### Impact of pneumococcal vaccination (NONE vs. PV)

As displayed in Table 4 and Table 5, there was no significant disease-related total cost difference for individuals vaccinated against pneumococci compared to unvaccinated individuals, neither in 2015 (− €87.81 [95% CI − €464.81; €289.19], RDiff -14.42%) nor in 2016 (− €73.37 [95% CI − €468.99; €322.26], RDiff -11.26%). However, we observed lower costs for outpatient care in 2015 of − €9.43 ([95% CI − €17.56; − €1.30], RDiff -26.75%) and in 2016 of − €12.93 ([95% CI − €25.37; − €0.48], RDiff -33.04%). We also observed very small but statistically significant lower costs for treatments with indication for respiratory disorders in 2015 of − €0.03 ([95% CI − €0.05; − €0.02], RDiff -92.62%).

**Table 4:** Costs of health care: effect of pneumococcal vaccination, per insurant in 2015 (NONE vs. PV)^a^

Direct costs	Adjusted mean costs in € (SE)		Adjusted mean cost difference in € (95% CI)	Relative difference (RDiff)	p value
	NONE (*n* = 72,867)	PV (*n* = 1136)			
Disease-related direct costs					
Total	608.95 (21.98)	521.14 (191.09)	− 87.81 (− 464.81; 289.19)	− 14.42%	0.65
Costs of disease-related hospital inpatient care	558.59 (21.61)	474.93 (190.38)	− 83.66 (− 459.21; 291.88)	− 14.98%	0.66
Costs of disease-related outpatient care	35.24 (2.70)	25.81 (3.15)	**− 9.43** (− 17.56; − 1.30)	**− 26.75%**	**0.02**
Costs of disease-related emergency services	14.44 (0.47)	8.86 (2.91)	− 5.57 (− 11.36; 0.21)	− 38.61%	0.06
Costs of disease-related antibiotics	0.52 (0.05)	0.24 (0.15)	− 0.28 (− 0.58; 0.03)	− 53.09%	0.08
Costs of disease-related inpatient rehabilitation	0.11 (0.06)	0.0000 (0.0000)	− 0.11 (− 0.23; 0.00004)	− 100.00%	0.05
Costs of treatments with indication for respiratory disorders	0.04 (0.01)	0.003 (0.003)	**− 0.03** (− 0.05; − 0.02)	**− 92.62%**	** ≤ 0.01**
Costs of antivirals	0.02 (0.004)	0.06 (0.04)	0.04 (− 0.04; 0.12)	252.68%	0.32
Total direct costs					
Total	7325.04 (58.10)	6184.44 (465.06)	**− 1140.60** (− 2059.21; − 221.99)	**− 15.57%**	** ≤ 0.01**
Costs of hospital inpatient care	2535.10 (33.93)	2291.63 (332.26)	− 243.48 (− 898.11; 411.16)	− 9.60%	0.47
Costs of outpatient care	773.39 (10.18)	807.41 (47.70)	34.02 (− 61.58; 129.62)	4.40%	0.49

^a^Value rounded up to two decimal places or first identifiable digit

**Table 5:** Costs of health care: effect of pneumococcal vaccination, per insurant in 2016 (NONE vs. PV)^a^

Direct costs	Adjusted mean costs in € (SE)		Adjusted mean cost difference in € (95% CI)	Relative difference (RDiff)	*p* value
	NONE (*n* = 68,848)	PV (*n* = 1088)			
Disease-related direct costs					
Total	651.60 (24.55)	578.23 (200.35)	− 73.37 (− 468.99; 322.26)	− 11.26%	0.72
Costs of disease-related hospital inpatient care	598.72 (23.87)	527.71 (199.15)	− 71.02 (− 464.15; 322.12)	− 11.86%	0.72
Costs of disease-related outpatient care	39.12 (3.69)	26.19 (5.16)	**− 12.93** (− 25.37; -0.48)	**− 33.04%**	**0.04**
Costs of disease-related emergency services	12.55 (0.39)	10.46 (2.80)	− 2.08 (− 7.63; 3.46)	− 16.61%	0.46
Costs of disease-related antibiotics	0.44 (0.07)	0.30 (0.13)	− 0.14 (− 0.43; 0.15)	− 31.35%	0.35
Costs of disease-related inpatient rehabilitation	0.72 (0.25)	2.20 (2.21)	1.49 (− 2.86; 5.84)	207.98%	0.50
Costs of treatments with indication for respiratory disorders	0.05 (0.01)	0.30 (0.18)	0.25 (− 0.11; 0.61)	497.62%	0.17
Costs of antivirals	0.003 (0.001)	0.0000 (0.0000)	**− 0.003** (− 0,01; − 0.001)	**-100.00%**	** ≤ 0.01**
Total direct costs					
Total	7658.85 (62.37)	6287.64 (408.78)	**− 1371.22** (− 2181.70; − 560.73)	**-17.90%**	** ≤ 0.01**
Costs of hospital inpatient care	2654.44 (36.92)	2222.09 (256.54)	− 432.35 (− 940.36; 75.66)	− 16.29%	0.10
Costs of outpatient care	814.15 (11.27)	856.56 (53.41)	42.40 (− 64.58; 149.38)	5.21%	0.44

^a^Value rounded up to two decimal places or first identifiable digit

Significantly lower total costs were found in 2015 and 2016 (2015: − €1140.60 [95% CI − €2059.21; − €221.99], RDiff -15.57%; 2016: − €1371.22 [95% CI − €2181.70; − €560.73], RDiff − 17.90%, Table 4 and 5).

With regard to health care utilisation (Table S4 and S5, supplementary material), we found widely mixed results except for disease-related outpatient care and treatments that were not statistically significant in 2015 and 2016.

### Impact of influenza and pneumococcal vaccination (NONE vs. BOTH)

For individuals who received both vaccinations (see Table 6 and Table 7), only costs for antivirals were significantly lower in vaccinees of − €0.02 ([95% CI − €0.02; − €0.01], RDiff − 100%) in 2015 and of − €0.003 ([95% CI -0.01; -0.001], RDiff − 100%) in 2016. Costs for disease-related outpatient care were higher in both years, but not statistically significant (2015: €23.63 [95% CI − €4.86; €52.11], RDiff 67.04%; 2016: €7.84 [95% CI − €10.13; €25.81], RDiff 20.04%). Most other results varied between 2015 and 2016 and were also not statistically significant.

**Table 6:** Costs of health care: effect of influenza and pneumococcal vaccination, per insurant in 2015 (NONE vs. BOTH)^a^

Direct costs	Adjusted mean costs in € (SE)		Adjusted mean cost difference in € (95% CI)	Relative difference (RDiff)	*p* value
	NONE (*n* = 72,867)	BOTH (*n* = 3333)			
Disease-related direct costs					
Total	608.95 (21.98)	518.68 (77.00)	− 90.27 (-247.22; 66.67)	− 14.82%	0.26
Costs of disease-related hospital inpatient care	558.59 (21.61)	425.85 (74.95)	− 132.74 (− 285.63; 20.16)	− 23.76%	0.09
Costs of disease-related outpatient care	35.24 (2.70)	58.86 (14.28)	23.63 (− 4.86; 52.11)	67.04%	0.10
Costs of disease-related emergency services	14.44 (0.47)	13.85 (1.98)	− 0.59 (− 4.57; 3.39)	− 4.09%	0.77
Costs of disease-related antibiotics	0.52 (0.05)	0.55 (0.10)	0.03 (− 0.19; 0.26)	6.47%	0.77
Costs of disease-related inpatient rehabilitation	0.11 (0.06)	0.0000 (0.0000)	− 0.11 (− 0.23; 0.0004)	− 100.00%	0.05
Costs of treatments with indication for respiratory disorders	0.04 (0.01)	0.07 (0.05)	0.04 (− 0.07; 0.14)	101.61%	0.50
Costs of antivirals	0.02 (0.004)	0.0000 (0.0000)	**− 0.02** (− 0.02; − 0.01)	**-100.00%**	** ≤ 0.01**
Total direct costs					
Total	7325.04 (58.10)	7656.79 (307.82)	331.75 (− 282.23; 945.73)	4.53%	0.29
Costs of hospital inpatient care	2535.10 (33.93)	2194.71 (122.91)	**− 340.39** (− 590.30; − 90.48)	**− 13.43%**	** ≤ 0.01**
Costs of outpatient care	773.39 (10.18)	1079.37 (65.44)	**305.98** (176.17; 435.79)	**39.56%**	** ≤ 0.01**

^a^Value rounded up to two decimal places or first identifiable digit

**Table 7:** Costs of health care: effect of influenza and pneumococcal vaccination, per insurant in 2016 (NONE vs. BOTH)^a^

Direct costs	Adjusted mean costs in € (SE)		Adjusted mean cost difference in € (95% CI)	Relative difference (RDiff)	p value
	None (*n* = 68,848)	Both (*n* = 3094)			
Disease-related direct costs					
Total	651.60 (24.55)	779.88 (168.40)	128.28 (− 205.28; 461.85)	19.69%	0.45
Costs of disease-related hospital inpatient care	598.72 (23.87)	688.64 (165.94)	89.92 (− 238.67; 418.51)	15.02%	0.59
Costs of disease-related outpatient care	39.12 (3.69)	46.96 (8.39)	7.84 (− 10.13; 25.81)	20.04%	0.39
Costs of disease-related emergency services	12.55 (0.39)	11.07 (1.53)	− 1.47 (− 4.56; 1.61)	− 11.75%	0.35
Costs of disease-related antibiotics	0.44 (0.07)	4.15 (3.82)	3.70 (− 3.78; 11.19)	839.79%	0.33
Costs of disease-related inpatient rehabilitation	0.72 (0.25)	3.74 (3.73)	3.02 (− 4.31; 10.35)	422.09%	0.42
Costs of treatments with indication for respiratory disorders	0.05 (0.01)	0.04 (0.03)	-0.01 (− 0.07; 0.06)	− 10.88%	0.87
Costs of antivirals	0.003 (0.001)	0.0000 (0.0000)	**-0.003** (− 0.01; − 0.001)	**− 100.00%**	** ≤ 0.01**
Total direct costs					
Total	7658.85 (62.37)	7965.71 (336.11)	306.86 (− 363.18; 976.89)	4.01%	0.37
Costs of hospital inpatient care	2654.44 (36.92)	2462.73 (191.95)	− 191.72 (− 574.84; 191.41)	− 7.22%	0.33
Costs of outpatient care	814.15 (11.27)	1111.90 (67.08)	**297.75** (164.43; 431.07)	**36.57%**	** ≤ 0.01**

^a^Value rounded up to two decimal places or first identifiable digit

Table 6 and 7 display that there were statistically significant higher total costs for outpatient care in both years of follow-up (2015: €305.98 [95% CI €176.17; €435.79] RDiff 39.56%; 2016: €297.75 [95% CI €164.43; €431.07], RDiff 36.57%). Other total costs did not differ significantly between groups.

As shown in supplementary material Table S6 and S7, lower costs for antivirals were also observable in the number of antivirals’ prescriptions in 2015 (− 0.0004 [95% CI − 0.001; − 0.0002], RDiff -100%) and 2016 (− 0.0001 [95% CI − 0.0001; − 0.00002]; RDiff -100%).

Similarly, higher costs for outpatient care were also reflected in significantly higher disease-related outpatient cases in both years (2015: 0.03 [95% CI 0.01; 0.06], RDiff 12.76%; 2016: 0.04 [95% CI 0.01; 0.07], RDiff 17.59%).

## Discussion

### Summary of results

In our retrospective cohort study with real-world data, we found that influenza vaccination appeared as cost-saving in 2016, not in 2015, with lower total direct disease-related health care costs per patient of − €178.87 in vaccinated than in non-vaccinated individuals. Main source of cost savings was hospital inpatient care, followed by emergency services. Contrary, costs for disease-related outpatient care rose slightly in the first year after vaccination. These effects were also observable in disease-related health care utilisation and in total costs of health care. Pneumococcal vaccination tends to be also cost-saving in total disease-related costs, but without statistical significance in both years of follow-up. However, costs for disease-related outpatient care were significantly lower in pneumococci-vaccinated than in unvaccinated individuals. In contrast to other observational studies, which found additive vaccine effects in terms of reduced hospitalisation for influenza and pneumonia as well as disease-related costs [13, 25], we were unable to show such (statistically significant) beneficial cost effects when patients were vaccinated against both diseases.

### Comparison with results from other non-model-based cost studies

Our results are in line with the sparse literature on comparable data analyses: Nichol et al. found in their claims data study that influenza vaccination can save total (direct and indirect) costs of − $US 39.35 per vaccinated healthy person and of − $US 34.55 per vaccinated at-risk person at the age of 65–74 years [11]. Our results are also supported by the prospective study from Gasparini et al., which was based on primary data from 2000 and included vaccination costs and costs connected with the onset of influenza such as drug prescriptions and hospitalisation, showing overall cost-saving effects of − €110.20 per individual ≥ 65 years vaccinated against influenza compared to those unvaccinated [26]. In that study, the main source of cost savings was the five times higher hospitalisation costs for unvaccinated individuals [26]. Our result of lower hospitalisation costs for individuals vaccinated against influenza is also in line with the findings of Chiu et al. who reported five times lower influenza- and pneumonia-associated hospitalisations in vaccinated compared to unvaccinated individuals in a Taiwanese claims data analysis from 2002 to 2009 including citizens ≥ 65 years [27]. Remarkably, Chiu et al. also found higher outpatient visits in single years which, however, were not statistically significant [27]. In contrast to our results and the studies mentioned so far, Chang et al. found higher inpatient expenditure for those vaccinated against influenza compared to unvaccinated individuals [13]. There are multifold potential explanations for the differing results between Chang and us, but it should be noted that the authors themselves pointed out that their findings contradict the previous studies which demonstrated that influenza vaccination is associated with a reduction of hospitalisations for influenza, pneumonia, or respiratory illness [13].

For pneumococcal vaccinations, the direction of being cost-saving without statistical significance may be supported by the results of a Canadian study from Luca et al. using claims data from all hospitalizations in Ontario in 2005, estimating reduced hospitalisation costs of 47.5–66.0% due to pneumonia [12].

For the combined influenza and pneumococcal vaccination, we could not confirm additive results, other than Chang et al. who found reduced disease-related inpatient costs [13], as well as Kawakami et al. who found lower costs for all-cause pneumonia in their Japanese randomized-controlled trial from 2005 with a 2 years follow-up [28].

### Medical interpretation of heterogeneous results

Parallel to the decrease of health care utilisation, costs in hospital inpatient care, and costs in emergency care, we found an increase in outpatient care after influenza vaccination. This might reflect the phenomenon of attenuation of diseases, which leads to a shift from hospitalisation to outpatient treatment. This was also observed by Tessmer et al. as a reduction of complications such as severe pneumonia after influenza vaccination in individuals ≥ 18 years [29]. This theory is also supported by a Chinese randomized-controlled trial showing that participants had a higher risk of acute respiratory infections associated with confirmed non-influenza respiratory virus infection (RR, 4.40; 95% CI, 1.31–14.8) [30]. The authors interpreted this phenomenon as reduced immunity against non-specific respiratory infection for the benefit of immunity against influenza or expression of a temporary non-specific immunity after influenza virus infection [30].

Besides a vaccination-induced attenuation of related diseases, the decline in total and disease-related hospital inpatient care may further be explained by a decrease of non-infectious complications such as cardiovascular events, which were found to be reduced after influenza vaccination [31, 32]. As we did not analyse cardiovascular outcomes, we were unable to estimate the effect of influenza and pneumococcal vaccination on cardiovascular events which can be a useful purpose for further studies.

Chang et al. [13] and Kawakami et al. [28] enrolled study participants within one single influenza season. Thus, our rigorous inclusion criteria of a yearly influenza vaccination over 3 years which was applied to ensure a longitudinal study on effects in combination with pneumococcal vaccination could have generated outcomes other than one-season-studies.

The variation of results in all vaccination groups between the seasons might be explained by the high dependence of results on seasonally varying influenza virus strains (and influenza vaccine matches), as there is a high interdependence between influenza and the other respiratory diseases.

Similarly, heterogeneous results were noticeable in our analysis of vaccine effectiveness in reducing influenza, pneumonia, and sepsis incidence and 90-day mortality in influenza and/or pneumococcal vaccinated compared to unvaccinated individuals, based on the same patient cohort and adjustment algorithm [33]. Likewise, we had to conclude that finding medical explanations for the heterogeneous results is challenging and demands for further research [33].

### Strengths

We performed a large retrospective cohort study on influenza and pneumococcal vaccination effects on health care utilisation and costs of health care in 138,877 individuals ≥ 60 years in Germany. Using a community-based claims database that includes data on nearly all divisions of inpatient and outpatient care, we were able to analyse a broad set of disease-related and total health care utilisation parameters and costs from a health insurers’ perspective of vaccinated individuals compared to those unvaccinated in a follow-up period of 24 months after index vaccination. To yield unbiased effect estimates, the inclusion of all relevant confounding variables is required. The access to a large longitudinal database enabled us to adjust for many potentially confounding covariates. For example, effect estimates were adjusted for systematic differences like health care seeking behaviour, pre-treatment comorbidities, use of chronic care, and health service utilisation. This alleviates the risk of biases like healthy vaccinee bias or bias by indication [34, 35]

By our real-world analysis, we overcome the limitations of model-based cost analyses, which results react very sensitive to the assumed vaccine effectiveness that varies between regions and age groups. In contrast to this, we observed real-world vaccination effects in a large regional population naturally including herd immunity effects—to which (since assumed) results of health economic models have also found to be sensitive [36–40]. In contrast to health economic models, our analyses included a population-based unselected cohort, and the use of a health claims database made it possible to conduct a study on a complete record of inpatient and outpatient care covering a time frame of nine consecutive years, which is a major strength of our study.

### Limitations

Beside these strengths, our study has several limitations, in particular the retrospective design and the unknown validity of administrative data, which are generated for reimbursement and not for research purpose. Although we used complex adjustment for four groups including 236 variables on demographics, comorbidities, health seeking behaviour, as well as previous utilisation and diseases, we are unable to fully rule out residual bias that particularly applies for the bias by indication. As seen in other studies, claims data do not fully represent frailty or health seeking behaviour [41] and provide only limited information about severity of comorbidities. The heterogeneous results may also reflect systematic differences between the 2015 and 2016 cohort: it must be considered that the 2016 sample in our study is a subsample without individuals that died in 2015 (*n* = 10,044, 7.23%). The 2016 subsample is on average younger, less dependent on nursing care and slightly healthier and can thus build up a better immunity after vaccination. This might cause different effects in 2015 and 2016 in single outcomes. Furthermore, we had to exclude 70,823 individuals from the vaccination groups and thus from effect assessment, leaving 66,010 vaccinees included in the assessment. The reduction was a necessary rule to avoid biased estimation of vaccine effectiveness due to previous vaccinations.

### Generalizability

Naturally, it is essential to consider whether results translate into other settings. As the costs of health care services and vaccination in particular differ substantially from country to country, the concrete ranges of cost effects will vary between countries, as then, finally, do the cost effects of establishing vaccination programs [11]. Pure health care utilisation figures may be better comparable. Nevertheless, results are still not fully transferable to other countries as there may be further systems differences that affect vaccination results (i.e., vaccination coverage/non-coverage for other age groups than targeted by recommendations which may affect the general prevalence of influenza- and pneumococci-associated diseases in a country).

## Conclusion

In conclusion, the interpretation of our findings is challenging, but we were able to show that influenza and pneumococcal vaccination can be cost-saving in selective seasons and health care divisions. We found cost-saving effects for influenza vaccination in various divisions of inpatient and outpatient health care services in 2015 and 2016. Pneumococcal vaccination appears to be cost-saving especially in disease-related outpatient health care. Although we could not fully rule out residual bias, (net) cost savings shown by real-world data-based studies like ours that already include vaccination costs and herd effects can enlarge the body of evidence and motivate health insurers to engage in programs for increasing vaccination rates in the elderly.

## Supplementary Information

Below is the link to the electronic supplementary material.

Supplementary file1 (DOCX 292 kb)
