# Supplementary material for: The effect of influenza and pneumococcal vaccination in the elderly on health service utilisation and costs: a claims data-based cohort study
Source: Eur J Health Econ. 2021 Jul 20;23(1):67–80. doi: 10.1007/s10198-021-01343-8 (PMC8882088; doi:10.1007/s10198-021-01343-8)
Supplement: Supplementary file 1 — Supplementary file1 (DOCX 292 kb) [file 10198_2021_1343_MOESM1_ESM.docx]

Supplementary Material

**The effect of influenza and pneumococcal vaccination in the elderly on health service utilisation and costs: a claims data-based cohort study**

Josephine Storch1,2, Carolin Fleischmann-Struzek3, Norman Rose3, Thomas Lehmann4, Anna Mikolajetz5, Srikanth Maddela4, Mathias W Pletz6, Christina Forstner6,7, Ole Wichmann8, Julia Neufeind8, Monique Vogel1, Konrad Reinhart3,5,9, Horst Christian Vollmar1,10, Antje Freytag1 and the vaccination 60+ study group

1 Institute of General Practice and Family Medicine, Jena University Hospital, Bachstraße 18, 07743 Jena, Germany

2 International Graduate Academy, Institute for Health and Nursing Science, Medical Faculty, Martin Luther University Halle-Wittenberg, Magdeburger Straße 8, 06112 Halle (Saale), Germany

3 Center for Sepsis Control and Care, Jena University Hospital, Bachstraße 18, 07743 Jena, Germany

4 Center for Clinical Studies, Jena University Hospital, Salvador-Allende-Platz 27, 07747 Jena, Germany

5 Department for Anesthesiology and Intensive Care Medicine, Jena University Hospital, Am Klinikum 1, 07740 Jena, Germany,

6 Institute of Infectious Diseases and Infection Control, Jena University Hospital, Am Klinikum 1, 07747 Jena, Germany

7 Department of Medicine I, Division of Infectious Diseases and Tropical Medicine, Medical University of Vienna, Währinger Gürtel, 18-20, 1090 Vienna, Austria

8 Immunization Unit, Robert Koch Institute, Seestraße 10, 13353 Berlin, Germany

9 BIH Visiting Professor/Charité Foundation, Department of Anesthesiology and Intensive Care Medicine, Charité Universitätsmedizin Berlin, Charitéplatz 1, 10117 Berlin, Germany

10 Institute of General Practice and Family Medicine, Medical Faculty, Ruhr-University Bochum, Universitätsstraße 150, 44801 Bochum, Germany

**Vaccination60+ study group:**

Cornelia Betsch, Sarah Eitze, Regina Hanke, Wolfgang Hanke, Dorothee Heinemeier, Carolin Fleischmann-Struzek, Christina Forstner, Antje Freytag, Nora Katharina Küpke, Thomas Lehmann, Srikanth Maddela, Anna Mikolajetz, Inga Petruschke, Mathias W. Pletz, Anne Reinhardt, Konrad Reinhart, Norman Rose, Constanze Rossmann, Philipp Schmid, Josephine Storch, Kasia Suchecka, Monique Vogel, Horst Christian Vollmar, Winja Weber

**Corresponding author:**

Dr. Antje Freytag

Institute of General Practice and Family Medicine, Jena University Hospital, Jena, Germany

antje.freytag@med.uni-jena.de

Content

[Table S1 Definitions of pre-treatment covariates for inverse probability weighting 3](#_Toc45483250)

[Figure S1 Pairwise absolute standardized mean differences in all covariates used for propensity score estimation before (unadjusted) and after IPW (adjusted) 9](#_Toc45483251)

[Table S2 Health care utilisation: Effect of influenza vaccination, per insurant in 2015 (NONE vs. IV)a 10](#_Toc45483252)

[Table S3 Health care utilisation: Effect of influenza vaccination, per insurant in 2016 (NONE vs. IV)^a^ 11](#_Toc45483253)

[Table S4 Health care utilisation: Effect of pneumococcal vaccination, per insurant in 2015 (NONE vs. PV)^a^ 12](#_Toc45483254)

[Table S5 Health care utilisation: Effect of pneumococcal vaccination, per insurant in 2016 (NONE vs. PV)^a^ 13](#_Toc45483255)

[Table S6 Health care utilisation: Effect of influenza and pneumococcal vaccination, per insurant in 2015 (NONE vs. BOTH)^a^ 14](#_Toc45483256)

[Table S7 Health care utilisation: Effect of influenza and pneumococcal vaccination, per insurant in 2016 (NONE vs. BOTH)^a^ 15](#_Toc45483257)

# Table S1 Definitions of pre-treatment covariates for inverse probability weighting

| **Covariate** | **Operationalization (comment)** |
| --- | --- |
| **Socioeconomic status** |  |
| Age | Age on December 31, 2014. |
| Sex | Male or female on January 1, 2014 |
| Nationality | German or non-German on January 01, 2014 |
| Place of residence | Urban or partly urban or rural (according to zip code and area classification of the Federal Institute for Research on Building, Urban Affairs and Spatial Development) |
| Employment | Coded “1” if the individual was employed according to insurance status on January 01, 2014, otherwise coded “0”. |
| Socioeconomic hardship status | Coded “1” if individual had socioeconomic hardship status on December 31, 2013, otherwise coded “0”. |
| Disability pension | Coded “1” if individual received disability pension on December 31, 2013, otherwise coded “0”. |
| **Health Care Use** |  |
| Health check-up | Regular health check ups are offered by statuary health insurances in Germany every two years. Participation was assessed using the following codes according to the Doctors' Fee Scale within the Statutory Health Insurance Scheme [Einheitlicher Bewertungsmaßstab, EBM]: 01730, 01731, 01732, 01733, 01734, 01734M, 01735, 01740, 01740M, 01741, 01741M, 01742, 01742M, 01743, 01743M, 01745, 01745M, 01746, 01746M, 99380, 99381, 99382, 99383, 99384, 99385 |
| Disease management program | Coded “1” if participation in at least one or more of the following disease management programs: coronary heart disease, diabetes mellitus, asthma, breast cancer or chronic obstructive pulmonary disease on December 31, 2013, otherwise coded “0”. |
| GP-centred healthcare program | Coded “1” if participation in GP-centered healthcare program on December 31, 2014, otherwise coded “0”. |
| Nursing care level | 0 (no nursing care level), 1 (nursing care level 1), 2 (nursing care level 2), 3 (nursing care level 3), 4 (nursing care level 4), 5 (nursing care level 4 with hardship status) on December 31, 2013 |
| Nursing home residence | Living in nursing home on December 2013 |
| Number of hospitalizations | Number of hospitalizations in 2013 |
| Hospital length of stay | Cumulative hospital length of stay in days in 2013 |
| Mechanical ventilation hours | Hours on in-hospital mechanical ventilation in 2013 |
| GP outpatient visits | Number of GP outpatient visits in 2013 |
| Specialist outpatient visits | Number of specialist outpatient visits in 2013 |
| Drug prescriptions | Number of different medications according to distinct ATC codes (Anatomical Therapeutic Chemical / Defined Daily Dose Classification codes) in 2013 |
| Prescription of antiviral medication | Coded “1” if individual had at least one prescription of an antiviral medication according to ATC codes J05AC, J05AH in 2013 |
| **Previous occurrence of target diseases** | Occurrence of target diseases was identified according to primary or secondary hospital discharge ICD-10-German Modification diagnoses or outpatient diagnoses in 2012, 2013 |
| Influenza-like illness | ICD-10-GM: J09, J10, J11 |
| Pneumonia | ICD-10-GM: J12, J13, J14, J15, J16, J18, U6900 |
| IPD | ICD-10-GM: A403, G001, J86, B953 |
| Sepsis | ICD-10-GM: A021, A200, A207, A217, A227, A241, A267, A282, A327, A391, A392, A393, A394, A40, A41, A427, A483, A499, A548, B007, B376, B377, B49, R650, R651, R572 |
| **Comorbidities** |  |
| German Hierarchical Morbidity Groups (HMGs) | Hierarchical morbidity groups in the morbidity-based risk adjustment scheme for statutory health insurances in Germany are calculated based on age, gender, disability pension, inpatient and outpatient diagnoses and medications for the purpose of risk structure compensation. For further information see documentation “Festlegung nach §31 Abs. 4 RSAV für das Ausgleichsjahr 2014” by the German Federal (Social) Insurance Office: https://www.bundesversicherungsamt.de/fileadmin/redaktion/Risikostrukturausgleich/Festlegungen/AJ_2014/Festlegung_Klassifikation_AJ2014.zip (last access on September 17, 2019)  HMG001, HMG003, HMG004, HMG005, HMG015, HMG016, HMG017, HMG019, HMG020, HMG021, HMG022, HMG023, HMG024, HMG025, HMG026, HMG027, HMG031, HMG032, HMG033, HMG034, HMG035, HMG039, HMG040, HMG041, HMG042, HMG043, HMG044, HMG046, HMG047, HMG049, HMG051, HMG052, HMG053, HMG054, HMG055, HMG056, HMG057, HMG058, HMG060, HMG071, HMG072, HMG073, HMG074, HMG075, HMG077, HMG078, HMG079, HMG080, HMG081, HMG083, HMG084, HMG086, HMG087, HMG088, HMG089, HMG091, HMG092, HMG095, HMG096, HMG099, HMG100, HMG101, HMG103, HMG104, HMG105, HMG106, HMG108, HMG110, HMG112, HMG130, HMG131, HMG132, HMG133, HMG134, HMG136, HMG138, HMG144, HMG146, HMG147, HMG149, HMG152, HMG157, HMG158, HMG160, HMG162, HMG163, HMG164, HMG165, HMG166, HMG169, HMG170, HMG174, HMG175, HMG176, HMG177, HMG184, HMG198, HMG199, HMG200, HMG202, HMG203, HMG204, HMG205, HMG207, HMG208, HMG209, HMG210, HMG211, HMG212, HMG213, HMG214, HMG215, HMG216, HMG217, HMG218, HMG219, HMG220, HMG223, HMG225, HMG226, HMG227, HMG228, HMG230, HMG231, HMG232, HMG233, HMG234, HMG235, HMG237, HMG251, HMG252, HMG253, HMG254, HMG255, HMG257, HMG258, HMG260, HMG261, HMG262, HMG263, HMG264, HMG265, HMG266, HMG267, HMG268, HMG269, HMG270, HMG271, HMG272, HMG273, HMG274, HMG275, HMG276, HMG277, HMG278, HMG279, HMG280, HMG283, HMG286, HMG287, HMG288, HMG289, HMG290, HMG291, HMG292, HMG293, HMG294, HMG295, HMG296, HMG297, HMG298, HMG299, HMG300, HMG301 |
| Charlson Comorbidity Score | Based on primary or secondary hospital discharge or outpatient diagnoses in 2013. Charlson Comorbidity-Score was calculated as described by Cui et al. [1]. |
| Elixhauser-Van Walraven.Score | Based on primary or secondary hospital discharge or outpatient ICD-10-GM diagnoses in 2013. Elixhauser-Score was calculated as described by Van Walraven et al. [2]. |
| **Other categories** (as residual categories) | To avoid colinearity, a comparison between different comorbidity indices was made on the level of ICD-codes. We included only codes for underlying chronic conditions as well as Charlson and Elixhauser comorbidity codes that were not included in the HMGs. Remaining codes were included in the following categories (as residual categories). |
| **Individuals with underlying chronic disease** (as residual categories) | Based on the recommendation for influenza and pneumococcal vaccination from the Standing Committee on Vaccination for Germany [3], we categorized individuals with underlying chronic conditions for which these vaccinations are recommended. For this we used primary or secondary hospital discharge or outpatient ICD-10-GM diagnoses in 2013. |
| - Immunodeficiency/  immunosuppression | ICD-10-GM: C73, D56, D57, D58, D70, D71, D72, D730, D731, D732, D735, D738, D739, D74, D75, D77, D80, D81, D82, D83, D84, D89, D90, M05, M06, M08, M123, M30, M31, M32, M33, M34, M350, M351, M352, M353, M354, M355, M356, M360, M45, Q890, Q899, Z08, Z510, Z511, Z512 |
| - Chronic cardiovascular disease | ICD-10-GM: I09, I52, I71 |
| - Chronic lung disease | ICD-10-GM: D860, D862, J68, J70, J82, P27 |
| - Chronic kidney disease | ICD-10-GM: I722, I823, N02, N13, Q60 |
| - Chronic metabolic disease | ICD-10-GM: E05, E15, E16, E78, E90, G3181, T383 |
| - Chronic liver disease | ICD-10-GM: B15, B16, B17, B19, I864, K750, K751, K754, K758, K759 |
| - Chronic neurological disease | ICD-10-GM: G14, G70, H34, I68 |
| **Charlson Comorbidities** (as residual categories) | Based on the code definition of Schwarzkopf et al. [4] Charlson Comorbidities were generated using primary or secondary hospital discharge or outpatient diagnose in 2013. |
| - Dementia | ICD-10-GM: F000, F001, F002, F009, F010, F011, F012, F013, F018, F019, F020, F021, F022, F023, F024, F028, F03, F051, G300, G301, G308, G309, G311 |
| - Peptic ulcer disease | ICD-10-GM: K250, K251, K252, K253, K254, K255, K256, K257, K259, K260, K261, K262, K263, K264, K265, K266, K267, K269, K270, K271, K272, K273, K274, K275, K276, K277, K279, K280, K281, K282, K283, K284, K285, K286, K287, K289 |
| - Cerebrovascular disease | ICD-10-GM: G4502, G4503, G4509, G4512, G4513, G4519, G4522, G4523, G4529, G4533, G4539, G4542, G4543, G4549, G4582, G4583, G4589, G4592, G4593, G4599, G468, H340, I650, I651, I652, I653, I658, I659, I660, I661, I662, I663, I664, I668, I669, I670, I6710, I6711, I672, I673, I675, I676, I677, I6788, I679, I680, I681, I682, I688 |
| - Any malignancy | ICD-10-GM: C430, C431, C432, C433, C434, C435, C436, C437, C438, C439, C750, C754, C755, C758, C759 |
| **Elixhauser Comorbidities** (as residual categories) | Based on the code definition of Schwarzkopf et al. [4] Elixhauser Comorbidities were generated using primary or secondary hospital discharge or outpatient diagnose in 2013. |
| - Blood loss anemia | ICD-10-GM: D500 |
| - Deficiency anemia | ICD-10-GM: D508, D509, D510, D511, D512, D513, D518, D519, D520, D521, D528, D529, D530, D531, D532, D538, D539 |
| - Weight loss | ICD-10-GM: E40, E41, E42, E43, E440, E441, E45, E46, R634, R64 |
| - Alcohol abuse | ICD-10-GM: E52, K292, T510, T511, T512, T513, T518, T519, Z502 |
| - Cardiac arrhythmias | ICD-10-GM: I441, I442, I443, I456, I459, I471, I479, I491, I492, I493, I494, I495, I498, I499, R000, R001, R008, Z450, Z4500, Z4501, Z4502, Z4508, Z950 |
| - Congestive heart failure | ICD-10-GM: I099, P290 |
| - Chronic pulmonary disease | ICD-10-GM: J40, J410, J411, J418, J42, J450, J451, J458, J459, J46, J60, J61, J620, J628, J630, J631, J632, J633, J634, J635, J638, J64, J65, J660, J661, J662, J668, J670, J671, J672, J673, J674, J675, J676, J677, J678, J679, J684, J701, J703 |
| - Depression | ICD-10-GM: F432 |
| - Fluid and electrolyte disorders | ICD-10-GM: E86, E870, E871, E872, E873, E874, E875, E876, E877, E878 |
| - Hypothyroidism | ICD-10-GM: E000, E001, E002, E009, E010, E011, E012, E018, E02, E030, E031, E032, E033, E034, E035, E038, E039 |
| - Liver disease | ICD-10-GM: I864, K700, K701, K709, K711, K713, K714, K715, K720, K7271, K7272, K7273, K7274, K7279, K729, K760, K762, K763, K764, K765, K766, K768, K769 |
| - Other neurological disorders | ICD-10-GM: G210, G312, G3181, G3182, G3188, G319, G320, G328, G934, R470, R560, R568 |
| - Leucemia | ICD-10-GM: C91, C92, C93, C94, C95 |
| - Obesity | ICD-10-GM: E6600, E6609, E6610, E6619, E6620, E6629, E6680, E6689, E6690, E6699 |
| - Paralysis | ICD-10-GM: G041 |
| - Peripheral vascular disorder | ICD-10-GM: I790, I792, K551, K5581, K5582, K5588, K559, Z9580, Z9581, Z9588, Z959 |
| - Rheumatoid arthritis/collagen vascular diseases | ICD-10-GM: L940, L941, L943, M1200, M1201, M1202, M1203, M1204, M1205, M1206, M1207, M1208, M1209, M461 |
| - Valvular disease | ICD-10-GM: A520, I091, I098, Z952, Z953, Z954 |
| Immobility | ICD-10-GM: R263 |
| Smoking | ICD-10-GM: F172 |
| Down Syndrom | ICD-10-GM: Q90 |

# Figure S1 Pairwise absolute standardized mean differences in all covariates used for propensity score estimation before (unadjusted) and after IPW (adjusted)


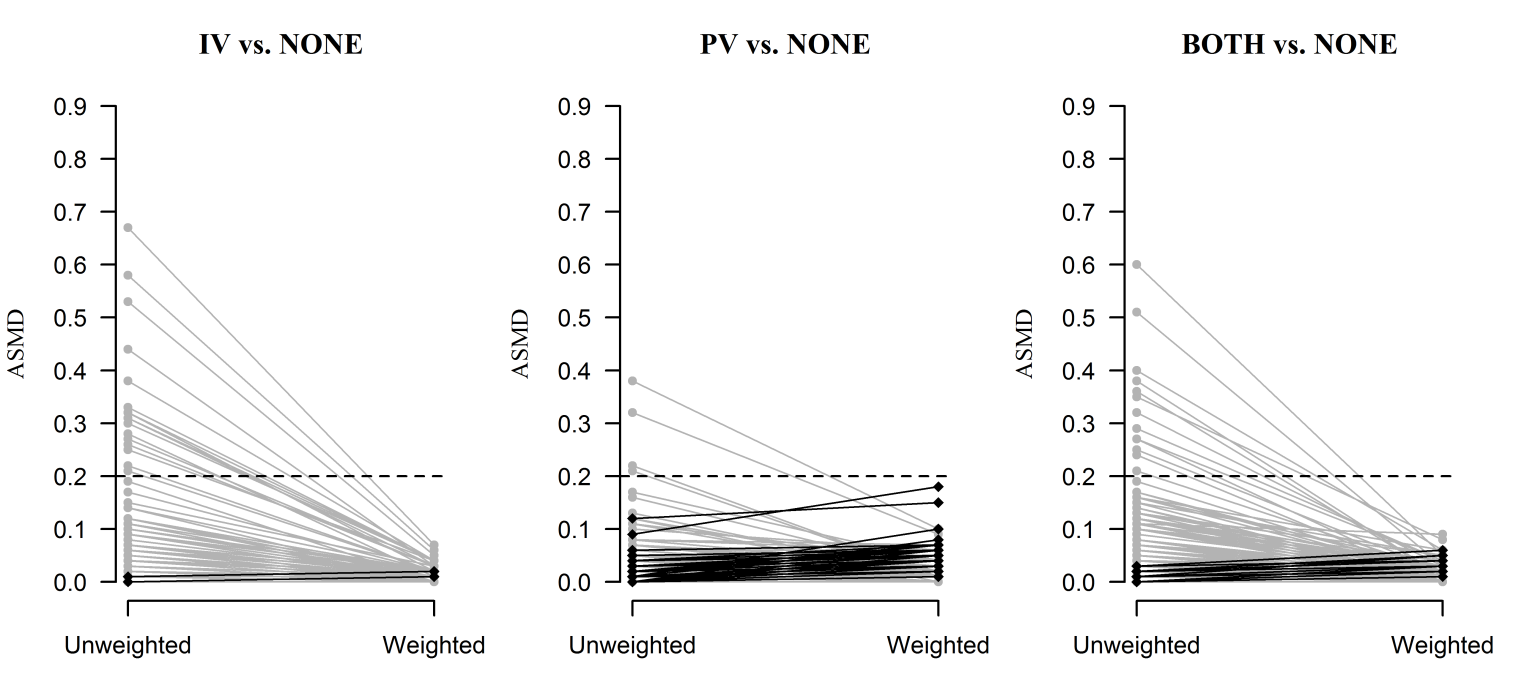


# Table S2 Health care utilisation: Effect of influenza vaccination, per insurant in 2015 (NONE vs. IV)a

| **Disease-related health care utilisation** | **Adjusted mean**  **in n (SE)** | | **Adjusted mean difference**  **(95% CI)** | **Relative Difference (RDiff)** | **p-value** |
| --- | --- | --- | --- | --- | --- |
|  | **NONE**  **(n=72,867)** | **IV**  **(n=61,541)** |  |  |  |
| Disease-related hospital inpatient care (billed cases) | 0.07 (0.001) | 0.06 (0.001) | **-0.005** (-0.01; -0.001) | **-7.45%** | **≤0.01** |
| Disease-related outpatient care (billed cases) | 0.26 (0.003) | 0.28 (0.003) | **0.03** (0.02; 0.04) | **11.21%** | **≤0.01** |
| Disease-related emergency services (billed cases) | 0.06 (0.002) | 0.05 (0.002) | **-0.01** (-0.01; -0.002) | **-10.39%** | **≤0.01** |
| Number of disease-related antibiotics’ prescriptions | 0.02 (0.001) | 0.02 (0.001) | **-0.003** (-0.005; -0.0004) | **-13.36%** | **0.02** |
| Number of disease-related prescribed daily defined doses of antibiotics | 0.18 (0.01) | 0.16 (0.01) | -0.01 (-0.04; 0.01) | -7.00% | 0.30 |
| Disease-related inpatient rehabilitation (billed cases) | 0.0001 (0.00003) | 0.0001 (0.00005) | 0.0001 (-0.00004; 0.0002) | 97.63% | 0.25 |
| Number of treatments with indication for respiratory disorders | 0.001 (0.0001) | 0.001 (0.0002) | **0.0005** (0.0002; 0.001) | **94.80%** | **≤0.01** |
| Number of antivirals’ prescriptions | 0.0004 (0.0001) | 0.0002 (0.0001) | -0.0002 (-0.0004; 0.00003) | -47.62% | 0.08 |
| Number of prescribed daily defined doses of antivirals | 0.002 (0.0005) | 0.001 (0.0003) | -0.001 (-0.002; 0.00002) | -51.31% | 0.06 |
| **Total health care utilisation** |  |  |  |  |  |
| Hospital inpatient care (billed cases) | 0.63 (0.01) | 0.57 (0.01) | **-0.05** (-0.07; -0.04) | **-8.29%** | **≤0.01** |
| Outpatient care (billed cases) | 10.48 (0.03) | 11.73 (0.04) | **1.26** (1.17; 1.35) | **12.00%** | **≤0.01** |
| ^a^ Value rounded up to two decimal places or first identifiable digit. | | | | | |

# Table S3 Health care utilisation: Effect of influenza vaccination, per insurant in 2016 (NONE vs. IV)^a^

| **Disease-related health care utilisation** | **Adjusted mean**  **in n (SE)** | | **Adjusted mean difference**  **(95% CI)** | **Relative Difference (RDiff)** | **p-value** |
| --- | --- | --- | --- | --- | --- |
|  | **NONE**  **(n=68,848)** | **IV**  **(n=55,803)** |  |  |  |
| Disease-related hospital inpatient care (billed cases) | 0.06 (0.001) | 0.05 (0.002) | **-0.01** (-0.02; -0.01) | **-18.58%** | **≤0.01** |
| Disease-related outpatient care (billed cases) | 0.23 (0.003) | 0.25 (0.003) | **0.02** (0.01; 0.03) | **9.62%** | **≤0.01** |
| Disease-related emergency services (billed cases) | 0.05 (0.001) | 0.04 (0.002) | **-0.01** (-0.01; -0.003) | **-15.99%** | **≤0.01** |
| Number of disease-related antibiotics’ prescriptions | 0.01 (0.001) | 0.01 (0.001) | -0.001 (-0.003; 0.001) | -8.44% | 0.24 |
| Number of disease-related prescribed daily defined doses of antibiotics | 0.14 (0.01) | 0.13 (0.01) | -0.01 (-0.03; 0.01) | -6.97% | 0.36 |
| Disease-related inpatient rehabilitation (billed cases) | 0.0002 (0.0001) | 0.0002 (0.0001) | -0.0001 (-0.0003; 0.0001) | -27.51% | 0.52 |
| Number of treatments with indication for respiratory disorders | 0.001 (0.0001) | 0.001 (0.0001) | 0.0002 (-0.0001; 0.001) | 31.18% | 0.24 |
| Number of antivirals’ prescriptions | 0.0001 (0.00003) | 0.0001 (0.00004) | 0.00005 (-0.0001; 0.0002) | 55.58% | 0.40 |
| Number of prescribed daily defined doses of antivirals | 0.0004 (0.0002) | 0.001 (0.0002) | 0.0002 (-0.0003; 0.001) | 55.58% | 0.40 |
| **Total health care utilisation** |  |  |  |  |  |
| Hospital inpatient care (billed cases) | 0.62 (0.01) | 0.53 (0.01) | **-0.09** (-0.10; -0.07) | **-14.09%** | **≤0.01** |
| Outpatient care (billed cases) | 10.49 (0.03) | 11.98 (0.04) | **1.48** (1.39; 1.58) | **14.13%** | **≤0.01** |
| ^a^ Value rounded up to two decimal places or first identifiable digit. | | | | | |

# Table S4 Health care utilisation: Effect of pneumococcal vaccination, per insurant in 2015 (NONE vs. PV)^a^

| **Disease-related health care utilisation** | **Adjusted mean**  **in n (SE)** | | **Adjusted mean difference**  **(95% CI)** | **Relative Difference (RDiff)** | **p-value** |
| --- | --- | --- | --- | --- | --- |
|  | **NONE**  **(n=72,867)** | **PV**  **(n=1136)** |  |  |  |
| Disease-related hospital inpatient care (billed cases) | 0.07 (0.001) | 0.04 (0.01) | **-0.03** (-0.04; -0.01) | **-37.45%** | **≤0.01** |
| Disease-related outpatient care (billed cases) | 0.26 (0.003) | 0.26 (0.02) | 0.005 (-0.04; 0.05) | 1.86% | 0.84 |
| Disease-related emergency services (billed cases) | 0.06 (0.002) | 0.03 (0.01) | **-0.02** (-0.04; -0.002) | **-40.40%** | **0.03** |
| Number of disease-related antibiotics’ prescriptions | 0.02 (0.001) | 0.01 (0.004) | **-0.01** (-0.02; -0.002) | **-55.17%** | **0.02** |
| Number of disease-related prescribed daily defined doses of antibiotics | 0.18 (0.01) | 0.07 (0.03) | **-0.11** (-0.17; -0.04) | **-60.19%** | **≤0.01** |
| Disease-related inpatient rehabilitation (billed cases) | 0.0001 (0.00003) | 0.0000 (0.0000) | **-0.0001** (-0.0001; -0.000002) | **-100.00%** | **0.04** |
| Number of treatments with indication for respiratory disorders | 0.001 (0.0001) | 0.0002 (0.0002) | -0.0003 (-0.001; 0.0001) | -61.99% | 0.15 |
| Number of antivirals’ prescriptions | 0.0004 (0.0001) | 0.002 (0.001) | 0.002 (-0.001; 0.004) | 362.62% | 0.29 |
| Number of prescribed daily defined doses of antivirals | 0.002 (0.0005) | 0.01 (0.004) | 0.004 (-0.004; 0.01) | 176.60% | 0.37 |
| **Total health care utilisation** |  |  |  |  |  |
| Hospital inpatient care (billed cases) | 0.63 (0.01) | 0.55 (0.04) | -0.07 (-0.16; 0.01) | -11.71% | 0.08 |
| Outpatient care (billed cases) | 10.48 (0.03) | 11.4 (0.24) | **0.92** (0.44; 1.40) | **8.80%** | **≤0.01** |
| ^a^ Value rounded up to two decimal places or first identifiable digit. | | | | | |

# Table S5 Health care utilisation: Effect of pneumococcal vaccination, per insurant in 2016 (NONE vs. PV)^a^

| **Disease-related health care utilisation** | **Adjusted mean**  **in n (SE)** | | **Adjusted mean difference**  **(95% CI)** | **Relative Difference (RDiff)** | **p-value** |
| --- | --- | --- | --- | --- | --- |
|  | **NONE**  **(n=68,848)** | **PV**  **(n=1088)** |  |  |  |
| Disease-related hospital inpatient care (billed cases) | 0.06 (0.001) | 0.05 (0.01) | -0.01 (-0.03; 0.01) | -20.13% | 0.24 |
| Disease-related outpatient care (billed cases) | 0.23 (0.003) | 0.24 (0.02) | 0.01 (-0.04; 0.06) | 5.54% | 0.61 |
| Disease-related emergency services (billed cases) | 0.05 (0.001) | 0.05 (0.01) | -0.01 (-0.03; 0.02) | -10.04% | 0.68 |
| Number of disease-related antibiotics’ prescriptions | 0.01 (0.001) | 0.02 (0.01) | 0.001 (-0.01; 0.01) | 5.14% | 0.90 |
| Number of disease-related prescribed daily defined doses of antibiotics | 0.14 (0.01) | 0.12 (0.04) | -0.02 (-0.11; 0.07) | -13.17% | 0.68 |
| Disease-related inpatient rehabilitation (billed cases) | 0.0002 (0.0001) | 0.0003 (0.0003) | 0.00003 (-0.0005; 0.001) | 14.50% | 0.90 |
| Number of treatments with indication for respiratory disorders | 0.001 (0.0001) | 0.01 (0.003) | 0.01 (-0.0001; 0.01) | 833.63% | 0.05 |
| Number of antivirals’ prescriptions | 0.0001 (0.00003) | 0.0000 (0.0000) | **-0.0001** (-0.0001; -0.00002) | **-100.00%** | **≤0.01** |
| Number of prescribed daily defined doses of antivirals | 0.0004 (0.0002) | 0.0000 (0.0000) | **-0.0004** (-0.001; -0.0001) | **-100.00%** | **≤0.01** |
| **Total health care utilisation** |  |  |  |  |  |
| Hospital inpatient care (billed cases) | 0.62 (0.01) | 0.56 (0.04) | -0.06 (-0.14; 0.03) | -9.15% | 0.19 |
| Outpatient care (billed cases) | 10.49 (0.03) | 11.59 (0.24) | **1.09** (0.62; 1.57) | **10.43%** | **≤0.01** |
| ^a^ Value rounded up to two decimal places or first identifiable digit. | | | | | |

# Table S6 Health care utilisation: Effect of influenza and pneumococcal vaccination, per insurant in 2015 (NONE vs. BOTH)^a^

| **Disease-related health care utilisation** | **Adjusted mean**  **in n (SE)** | | **Adjusted mean difference**  **(95% CI)** | **Relative Difference (RDiff)** | **p-value** |
| --- | --- | --- | --- | --- | --- |
|  | **NONE**  **(n=72,867)** | **BOTH**  **(n=3333)** |  |  |  |
| Disease-related hospital inpatient care (billed cases) | 0.07 (0.001) | 0.06 (0.01) | -0.003 (-0.02; 0.01) | -4.50% | 0.65 |
| Disease-related outpatient care (billed cases) | 0.26 (0.003) | 0.29 (0.01) | **0.03** (0.01; 0.06) | **12.76%** | **0.02** |
| Disease-related emergency services (billed cases) | 0.06 (0.002) | 0.05 (0.01) | -0.01 (-0.02; 0.01) | -10.05% | 0.36 |
| Number of disease-related antibiotics’ prescriptions | 0.02 (0.001) | 0.02 (0.004) | 0.004 (-0.003; 0.01) | 22.22% | 0.27 |
| Number of disease-related prescribed daily defined doses of antibiotics | 0.18 (0.01) | 0.24 (0.04) | 0.06 (-0.02; 0.14) | 35.74% | 0.12 |
| Disease-related inpatient rehabilitation (billed cases) | 0.0001 (0.00003) | 0.0000 (0.0000) | **-0.0001** (-0.0001; -0.000002) | **-100.00%** | **0.04** |
| Number of treatments with indication for respiratory disorders | 0.001 (0.0001) | 0.001 (0.001) | 0.001 (-0.001; 0.002) | 119.66% | 0.36 |
| Number of antivirals’ prescriptions | 0.0004 (0.0001) | 0.0000 (0.0000) | **-0.0004** (-0.001; -0.0002) | **-100.00%** | **≤0.01** |
| Number of prescribed daily defined doses of antivirals | 0.002 (0.0005) | 0.0000 (0.0000) | **-0.002** (-0.003; -0.001) | **-100.00%** | **≤0.01** |
| **Total health care utilisation** |  |  |  |  |  |
| Hospital inpatient care (billed cases) | 0.63 (0.01) | 0.59 (0.02) | -0.04 (-0.08; 0.01) | -6.04% | 0.09 |
| Outpatient care (billed cases) | 10.48 (0.03) | 11.96 (0.13) | **1.49** (1.23; 1.75) | **14.19%** | **≤0.01** |
| ^a^ Value rounded up to two decimal places or first identifiable digit. | | | | | |

# Table S7 Health care utilisation: Effect of influenza and pneumococcal vaccination, per insurant in 2016 (NONE vs. BOTH)^a^

| **Disease-related health care utilisation** | **Adjusted mean**  **in n (SE)** | | **Adjusted mean difference**  **(95% CI)** | **Relative Difference (RDiff)** | **p-value** |
| --- | --- | --- | --- | --- | --- |
|  | **NONE**  **(n=68,848)** | **BOTH**  **(n=3094)** |  |  |  |
| Disease-related hospital inpatient care (billed cases) | 0.06 (0.001) | 0.06 (0.01) | -0.01 (-0.02; 0.01) | -9.47% | 0.41 |
| Disease-related outpatient care (billed cases) | 0.23 (0.003) | 0.27 (0.01) | **0.04** (0.01; 0.07) | **17.59%** | **≤0.01** |
| Disease-related emergency services (billed cases) | 0.05 (0.001) | 0.04 (0.01) | -0.01 (-0.02; 0.01) | -12.13% | 0.32 |
| Number of disease-related antibiotics’ prescriptions | 0.01 (0.001) | 0.02 (0.01) | 0.01 (-0.01; 0.02) | 43.55% | 0.35 |
| Number of disease-related prescribed daily defined doses of antibiotics | 0.14 (0.01) | 0.24 (0.08) | 0.09 (-0.06; 0.25) | 66.55% | 0.22 |
| Disease-related inpatient rehabilitation (billed cases) | 0.0002 (0.0001) | 0.002 (0.002) | 0.002 (-0.002; 0.01) | 736.27% | 0.38 |
| Number of treatments with indication for respiratory disorders | 0.001 (0.0001) | 0.001 (0.0004) | -0.0001 (-0.001; 0.001) | -9.41% | 0.88 |
| Number of antivirals’ prescriptions | 0.0001 (0.00003) | 0.0000 (0.0000) | **-0.0001** (-0.0001; -0.00002) | **-100.00%** | **≤0.01** |
| Number of prescribed daily defined doses of antivirals | 0.0004 (0.0002) | 0.0000 (0.0000) | **-0.0004** (-0.001; -0.0001) | **-100.00%** | **≤0.01** |
| **Total health care utilisation** |  |  |  |  |  |
| Hospital inpatient care (billed cases) | 0.62 (0.01) | 0.57 (0.02) | **-0.06** (-0.10; -0.01) | **-8.94%** | **≤0.01** |
| Outpatient care (billed cases) | 10.49 (0.03) | 12.41 (0.13) | **1.92** (1.65; 2.18) | **18.28%** | **≤0.01** |
| ^a^ Value rounded up to two decimal places or first identifiable digit. | | | | | |

References

1. Cui, Y., Wang, T., Bao, J., Tian, Z., Lin, Z., Chen, D.: Comparison of Charlson's weighted index of comorbidities with the chronic health score for the prediction of mortality in septic patients. Chinese medical journal 127, 2623–2627 (2014)

2. van Walraven, C., Austin, P.C., Jennings, A., Quan, H., Forster, A.J.: A modification of the Elixhauser comorbidity measures into a point system for hospital death using administrative data. Medical care (2009). https://doi.org/10.1097/MLR.0b013e31819432e5

3. Robert Koch-Institut (RKI): Empfehlungen der Ständigen Impfkommission (STIKO) am Robert Koch-Institut/Stand: August 2014 34, 305-340 (2014)

4. Schwarzkopf, D., Fleischmann-Struzek, C., Rüddel, H., Reinhart, K., Thomas-Rüddel, D.O.: A risk-model for hospital mortality among patients with severe sepsis or septic shock based on German national administrative claims data. PloS one (2018). https://doi.org/10.1371/journal.pone.0194371.
